# Therapeutic Potential of Agmatine in Essential Tremor Through Regulation of Lingo‐1 and Inflammatory Pathways

**DOI:** 10.1002/brb3.70241

**Published:** 2025-01-08

**Authors:** Zeynab Pirmoradi, Zahra Esmaili, Mohsen Nakhaie, Kristi A. Kohlmeier, Mohammad Shabani, Moazamehosadat Razavinasab, Mehran Ilaghi

**Affiliations:** ^1^ Neuroscience Research Center, Institute of Neuropharmacology Kerman University of Medical Sciences Kerman Iran; ^2^ Gastroenterology and Hepatology Research Center, Institute of Basic and Clinical Physiology Sciences Kerman University of Medical Sciences Kerman Iran; ^3^ Department of Drug Design and Pharmacology, Faculty of Health Sciences University of Copenhagen Copenhagen Denmark; ^4^ Department of Physiology, Medical School Kerman University of Medical Sciences Kerman Iran

**Keywords:** agmatine, essential tremor, Lingo‐1, neuroinflammation

## Abstract

**Purpose:**

Essential tremor (ET) is a prevalent movement disorder, yet current therapeutic options remain limited. Emerging evidence implicates leucine‐rich repeat and immunoglobulin‐like domain‐containing protein (Lingo‐1) and neuroinflammation in the pathophysiology of ET. This study aimed to investigate whether agmatine, a biogenic amine neuromodulator attenuates tremors and modulates the expression of Lingo‐1 and proinflammatory markers in a rodent model of ET.

**Methods:**

Tremor was induced in male Swiss Webster mice through intraperitoneal injections of harmaline (10 mg/kg) on Days 1, 3, and 5 of the study. During the same period, agmatine (40 mg/kg) was administered for 5 consecutive days. Behavioral assessments of tremor severity, gait, balance, muscular strength, locomotion, anxiety‐like behavior, and memory were conducted. Moreover, Lingo‐1 and interleukin (IL)‐6 gene expression was examined in the cerebellum using real‐time polymerase chain reaction (RT‐PCR).

**Findings:**

Our findings demonstrated that agmatine administration significantly reduced tremors, ameliorated anxiety‐like behaviors, and attenuated harmaline‐induced locomotor deficits. At the molecular level, agmatine treatment significantly suppressed the overexpression of Lingo‐1 elicited by harmaline. Moreover, IL‐6 expression was attenuated to an extent comparable to control levels.

**Conclusions:**

Collectively, this study provides the first evidence that agmatine dampens tremor severity, improves behavioral outcomes, and modulates key pathways implicated in ET pathogenesis in a rodent model. The ability of agmatine to normalize Lingo‐1 and IL‐6 expression suggests regulation of these pathways could underlie its neuroprotective action. These results suggest promise for agmatine as a prospective therapeutic agent in ET.

## Graphical abstract

Agmatine significantly reduced tremors, ameliorated anxiety‐like behaviors, and attenuated harmaline‐induced locomotor deficits. At the molecular level, agmatine significantly suppressed the overexpression of Lingo‐1 elicited by harmaline. Moreover, IL‐6 expression was attenuated to an extent comparable to control levels.

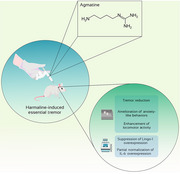

## 1 Introduction

Essential tremor (ET) is one of the most prevalent movement disorders, affecting almost 1% of individuals worldwide (Haubenberger and Hallett 2018). ET is primarily characterized by rhythmic and involuntary oscillations of a 4–12 Hz frequency, which affect various body parts, particularly the hands and arms. Additional presentations, including gait ataxia, intention tremor, and cognitive impairments, have also been reported (Rao and Louis 2019; Cartella et al. 2022). ET pathology is believed to be due to dysfunction in cerebellar and cerebellothalamocortical circuits (Welton et al. 2021).

The utilization of animal models in ET has allowed the examination of potential mechanisms that underlie this disease. Action‐dependent tremors can be induced in animals by harmaline, an alkaloid metabolite derived from the plant *Peganum harmala* (Doskaliyev et al. 2021). Harmaline‐induced effects closely mirror the observed tremor patterns in humans and are due to disruption of the olivocerebellar pathway through induction of neuronal desynchronization and arrhythmicity in inferior olive activity (Handforth 2012; Lang and Handforth 2022). Harmaline‐induced tremor has been widely utilized to examine mechanisms underlying ET and is useful for preclinical screening of potential ET therapeutic agents (Handforth et al. 2023; Abbassian et al. 2024).

Although several mechanisms have been proposed in the pathophysiology of ET (Pan and Kuo 2022), the leucine‐rich repeat and immunoglobulin‐like domain containing 1 (Lingo‐1) gene has gained significant attention as a risk factor for ET (Z. Zhou, Sathiyamoorthy, and Tan 2012). A Lingo‐1 sequence variant has been linked with ET in numerous genome‐wide association studies (Wu et al. 2011; Jiménez‐Jiménez et al. 2012; Jiménez‐Jiménez et al. 2021). Furthermore, Lingo‐1 is upstream of the EGFR–PI3K–AKT signaling pathway, which plays an essential role in the pathogenesis of ET and affects the survival of Purkinje cells in the cerebellum (Clark et al. 2022). Heightened cerebellar Lingo‐1 expression in distal axonal processes of basket cells was associated with the formation of a thin network surrounding the axon segment of Purkinje cells in repository‐preserved ET brains (Kuo et al. 2013), highlighting a role for this pathway in the pathogenesis of human ET. Therefore, Lingo‐1 has been proposed as a potential therapeutic target in ET (Clark et al. 2022; Agundez et al. 2015). On the other hand, neuroinflammation has been proposed as another factor contributing to ET. Elevated levels of inflammatory markers, including IL‐6, are shown to be associated with the severity of ET and cognitive and behavioral outcomes, suggesting a contribution of neuroinflammation in the processes underlying ET (Muruzheva, Ivleva, et al. 2022; Muruzheva, Traktirov, et al. 2022). Accordingly, reducing neuroinflammatory responses represents another potential target for the management of symptom progression or severity of ET.

Traditional treatment options such as beta‐blockers and anticonvulsants often provide limited relief in ET and are often associated with undesirable side effects. Thus, novel approaches to understand and treat ET require exploration. Agmatine has gained attention in various neurological disorders, including those exhibiting neurodegeneration (Xu et al. 2018; Chandurkar et al. 2022). Agmatine is a biogenic amine biosynthesized from l‐arginine through the action of arginine decarboxylase (Uzbay 2012). This agent exhibits high‐affinity binding to imidazoline receptors and α_2_‐adrenoceptors. In addition, it functions as an antagonist of the *N*‐methyl‐d‐aspartate (NMDA) receptor and acts as a competitive inhibitor of nitric oxide synthase (NOS) (Gawali et al. 2017). Agmatine has exhibited neuroprotective properties and therapeutic potential in spinal cord injury (Dixit et al. 2018), traumatic brain injury (J. Y. Kim et al. 2015), and brain ischemia (Cui et al. 2012). Agmatine has also been shown to ameliorate the behavioral and cognitive deficits associated with prenatal stress (Hassanshahi et al. 2023a, 2023b) and chronic unpredictable stress (Gawali et al. 2017; Taksande et al. 2013). Furthermore, evidence suggests that agmatine possesses antinociceptive (Kotagale et al. 2013), anxiolytic (Gawali et al. 2017), antidepressive (Ozkartal et al. 2019), and anti‐inflammatory (J. M. Kim et al. 2016; Milosevic et al. 2022) properties.

To date, there is a scarcity of studies examining the effects of agmatine on ET and its underlying mechanisms. Moreover, agmatine's potential to target the Lingo‐1 pathway has not been evaluated. Accordingly, this study aims to investigate the potential of agmatine in alleviating ET symptoms, with a specific emphasis on its interaction with Lingo‐1 and its involvement in inflammatory pathways within the cerebellum.

## 2 Materials and Methods

### 2.1 Animals

Male Swiss Webster (SW) mice (aged 8 weeks old and weighing 20–25 g) were used as experimental subjects in this study. Animals were kept in cages of six with adequate access to food and water under standard environmental conditions (room temperature of 25 ± 2°C, 12/12 light/dark cycle). All experiments were conducted following the protocols of Kerman University of Medical Sciences Laboratory Animal Care and approved by the Institutional Review Board (IRB) of Kerman University of Medical Sciences (IRB code: IR.KMU.REC.1401.404).

### 2.2 Experimental Design and Drugs

Animals were randomly assigned to four groups (*n* = 8 in each group) as follows: control, harmaline, agmatine, and harmaline + agmatine.

**FIGURE 1:**
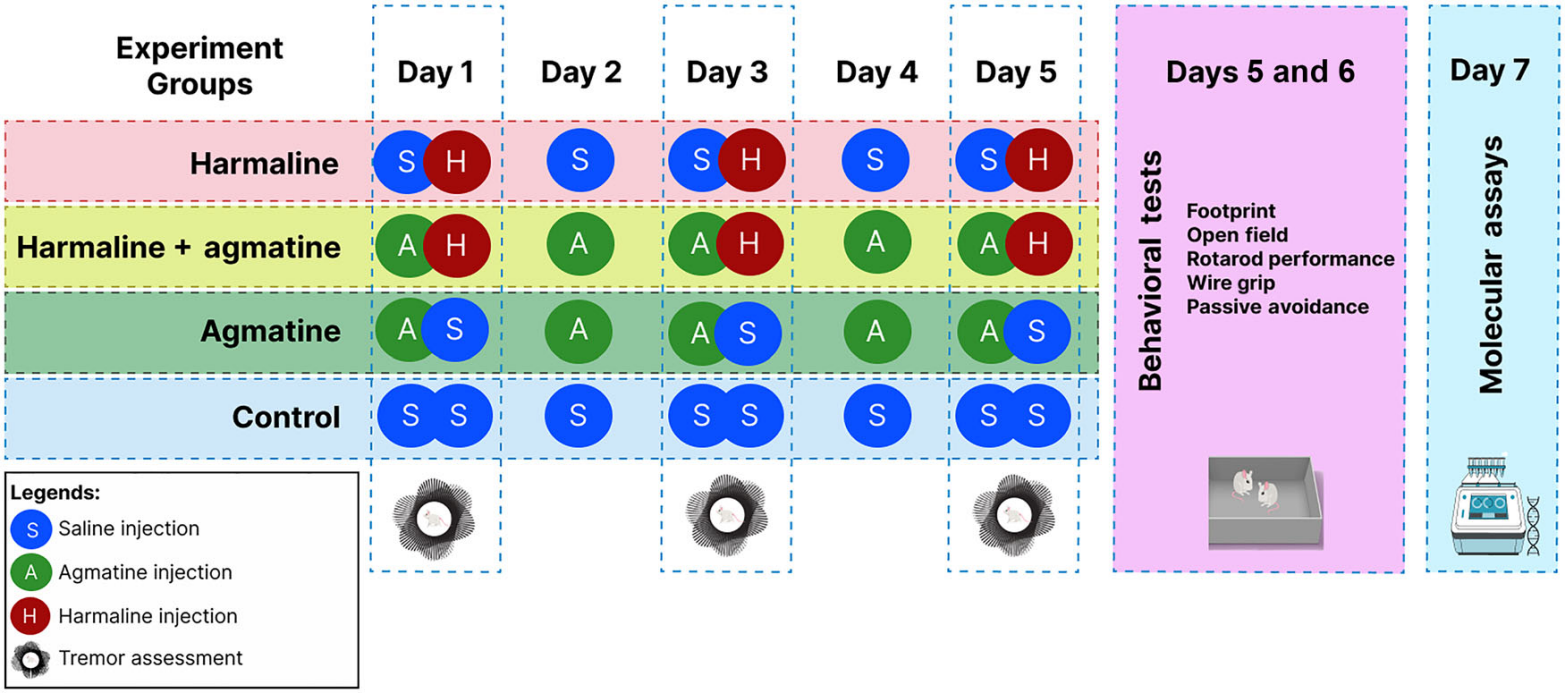
A timeline summarizing the study groups and timing of behavioral/molecular tests.

Harmaline was administered as harmaline hydrochloride dihydrate (10 mg/kg; H1392, Sigma, USA) intraperitoneally (ip) on the first, third, and fifth days of the study to induce ET (Pirmoradi et al. 2024). In the harmaline + agmatine group, in addition to the harmaline injections on the first, third, and fifth days, animals received agmatine (40 mg/kg, ip, lot number: MKCM5630, USA) for 5 consecutive days throughout the first to fifth days of study. In the days in which both agmatine and harmaline were administered, agmatine injections occurred 60 min before harmaline administration. The agmatine group received daily agmatine (40 mg/kg, ip) and the control group received daily ip saline injections for 5 days. Animals that only received a single drug agent also received saline 60 min before their injections on the first, third, and fifth days to ensure that all study groups received the same number of injections. Accordingly, the total number of injections in each group was 8 (Figure 1). Tremors were assessed on the first, third, and fifth days of the study. Other behavioral tasks were performed on the fifth and sixth days. Molecular assays were performed on the seventh day of the study. An overview of the study timeline is provided in Figure 1.

### 2.3 Behavioral Tests

The following behavioral tests were conducted on each group:

#### 2.3.1 Tremor Scoring

The occurrence and severity of tremors were evaluated by an observer who was blinded to the treatment assignment. The following quantitative tremor scoring was utilized to assess the severity of tremor 20 min after drug injections: 0: absence of tremor, 1: occasional tremor affecting just the head and neck, 2: intermittent tremor affecting all body parts, 3: persistent tremor involving all parts of the body, 4: severe tremor preventing the animal from standing and/or walking (Mohammadi et al. 2019).

#### 2.3.2 Footprint Test

The footprint test was conducted to evaluate elements of the walking pattern and gait of the animals. The animals' hind paws were dyed with a nontoxic ink. Animals were then allowed to walk in a 100 cm length × 10 cm width × 10 cm height Plexiglas tunnel leading to a dark cage. A strip of white absorbent paper measuring 100 cm × 10 cm was affixed to the bottom of the Plexiglas tube. The test had no time limit, and animals were allowed to walk to the end of the tunnel. The stride length was calculated by measuring the distance between the centers of each paw print on one side of the body. The hind paw stride width was calculated by measuring the distance between paw prints on either side of the body. Both the first and last footprints of each trial were excluded from the analysis (Vaziri et al. 2015).

#### 2.3.3 Open Field Test

To evaluate the locomotor activity and anxiety‐like behavior, the open field box, which was comprised of a square Plexiglas apparatus measuring 90 × 90 × 45 [H] cm and divided into 16 squares was utilized. The core zone consisted of 4 squares, whereas the peripheral zone encompassed 12 squares. Each animal was positioned in the center of the area at the beginning of the test session. Total distance moved (TDM) (cm), velocity (cm/s), number of grooming events, number of rearing episodes, and time spent in the central and peripheral zones (s) were recorded for 5 min. Activities were videotaped and analyzed offline using EthoVision software (version 7.1; Noldus Information Technology, the Netherlands). Between tests, the field was cleaned using a cotton cloth and alcohol (Gould, Dao, and Kovacsics 2009).

#### 2.3.4 Rotarod Performance Test

To evaluate the motor coordination and balance of each animal, an accelerated rotarod was employed. All animals were given a day of pretest training. Accordingly, mice became used to the rotarod device and learned what to do during pretraining. The process consisted of similar trials with a 5‐s ramp from 0 to a low rotational speed, followed by a steady velocity (17 rpm) for up to 40 s. The test procedure involved placing the animal on a rotating rod with speeds varying from 10 to 60 rpm. To determine the average time the animal remained on the rod, a time limit of 300 s and a 5 min break between three trials were implemented (Mohammadi et al. 2019; Pritchett and Mulder 2003).

#### 2.3.5 Wire Grip Test

The muscular strength of the animals was assessed using the wire grip test. In this setup, animals were suspended between two platforms utilizing a horizontal steel wire measuring 80 cm in length and 7 mm in diameter. The rodent maintained an upright position with its front feet together while clinging to the wire. Three sessions were conducted on each animal, with a 5‐min interval between sessions. The latency to fall in each session was measured (Jamwal, Singh, and Bansal 2017).

#### 2.3.6 Passive Avoidance Test

To assess passive avoidance learning and memory, a shuttle‐box apparatus was employed. This apparatus consisted of two chambers, one illuminated and the other in darkness separated by a grid door. The dark chamber's floor rods were connected to a shock generator. As a form of habituation, the mice were exposed to the apparatus 1 h before the test. Each mouse was put in the light chamber and was allowed 30 s to freely investigate the dark chamber. During the learning session, once the mouse entered the dark compartment, a single electric shock (0.5 mA, 2 s) was administered. The recorded parameter included the number of electric shocks administered to each animal as they sought to avoid entering the dark compartment. To evaluate memory, the mouse was situated in the light compartment 24 h after the learning session, and the door between the compartments was opened. The time taken to enter the dark chamber, known as step‐through latency (STL) was measured (Jarvik and Kopp 1967).

### 2.4 Molecular Assays

Twenty‐four hours after the final behavioral experiment, the animals were deeply anesthetized with isoflurane. Subsequently, saline and Buen fixative were perfused through the heart, and the brains were extracted. Cerebellar tissues were promptly excised. These tissues were rapidly frozen in liquid nitrogen and preserved at −80°C. Subsequently, RNA was isolated from cerebellum tissues using the RNA Kit (SinaPure‐RNA‐EX6031, Tehran Cavosh Clon, Iran). The concentration of the obtained RNAs was measured by a BioPhotometer (NanoDrop, N‐D 2000, Thermo Scientific, USA). The integrity of RNA was assessed by subjecting it to electrophoresis on an agarose gel (18 and 28 srRNA bands).

To explore the expression of Lingo‐1 and IL‐6, the extracted RNAs underwent cDNA synthesis using the AddScript cDNA Synthesis Kit (AddBio, Korea). In this reaction, 1–5 µg of each sample (based on concentration) was combined with 1 µL of 50 µM random hexamer, 13.4 µL of diethyl pyrocarbonate (DEPC) water, 1 µL of deoxynucleotide triphosphate (dNTPs) (10 mM), 0.5 µL of RNase inhibitor (20 units), and 1 µL of reverse transcriptase (200 units). The total volume of the reaction was 20 µL, and ncubation proceeded according to the specified temperature cycle. Synthesized cDNAs were preserved at −70°C until further analysis using real‐time polymerase chain reaction (PCR). Each reaction mixture comprised 12.5 µL of RealQ Plus 2x Master Mix, 0.5 mL of each primer, 10.5 µL of PCR‐grade H_2_O, and 1 µL of cDNA, resulting in a final volume of 25 µL. The PCR reaction was conducted with the following thermal cycling conditions: an initial denaturation step at 95°C for 15 min, followed by 40 cycles at 95°C for 15 s, 59°C for 30 s, and incubation at 72°C for 30 s. The total run time for the PCR process was approximately 2 h. Primers to amplify Lingo homologs were mLingo‐1For (5′‐TCTATCACGCACTGCAACCTGAC‐3′), and mLingo‐1Rev (5′‐ AGCATGGAGCCCTCGATTGTA‐3′). Primers for IL‐6 were IL‐6For (5′‐ AGACAGCCACTCACCTCTTCAG‐3′) and IL‐6Rev (5′‐TTCTGCCAGTGCCTCTTTGCTG‐3′). In addition, the glyceraldehyde 3‐phosphate dehydrogenase (GADPH) gene served as an internal control. The relative changes in the expression of target genes compared to a reference gene were determined using the 2^−ΔΔ^ *^C^* ^T^ method (Livak and Schmittgen 2001).

### 2.5 Statistical Analysis

GraphPad Prism 8 (GraphPad Software, USA) was used for statistical analysis and graph production. Data were tested for normal distribution according to the Kolmogorov–Smirnov test. Results found to be normally distributed (*p* > 0.05) were expressed as mean ± standard error of the mean (SEM) and analyzed using a one‐way ANOVA test. Otherwise, a nonparametric Kruskal–Wallis test was employed, and the data were reported using the median, with an interquartile range. *p* < 0.05 was considered statistically significant. To compare the means between different groups and time interactions in tremor score findings, a two‐way ANOVA followed by a Tukey's post hoc analysis was used.

## 3 Results

### 3.1 Agmatine Significantly Improved the Harmaline‐Induced Tremor

**FIGURE 2:**
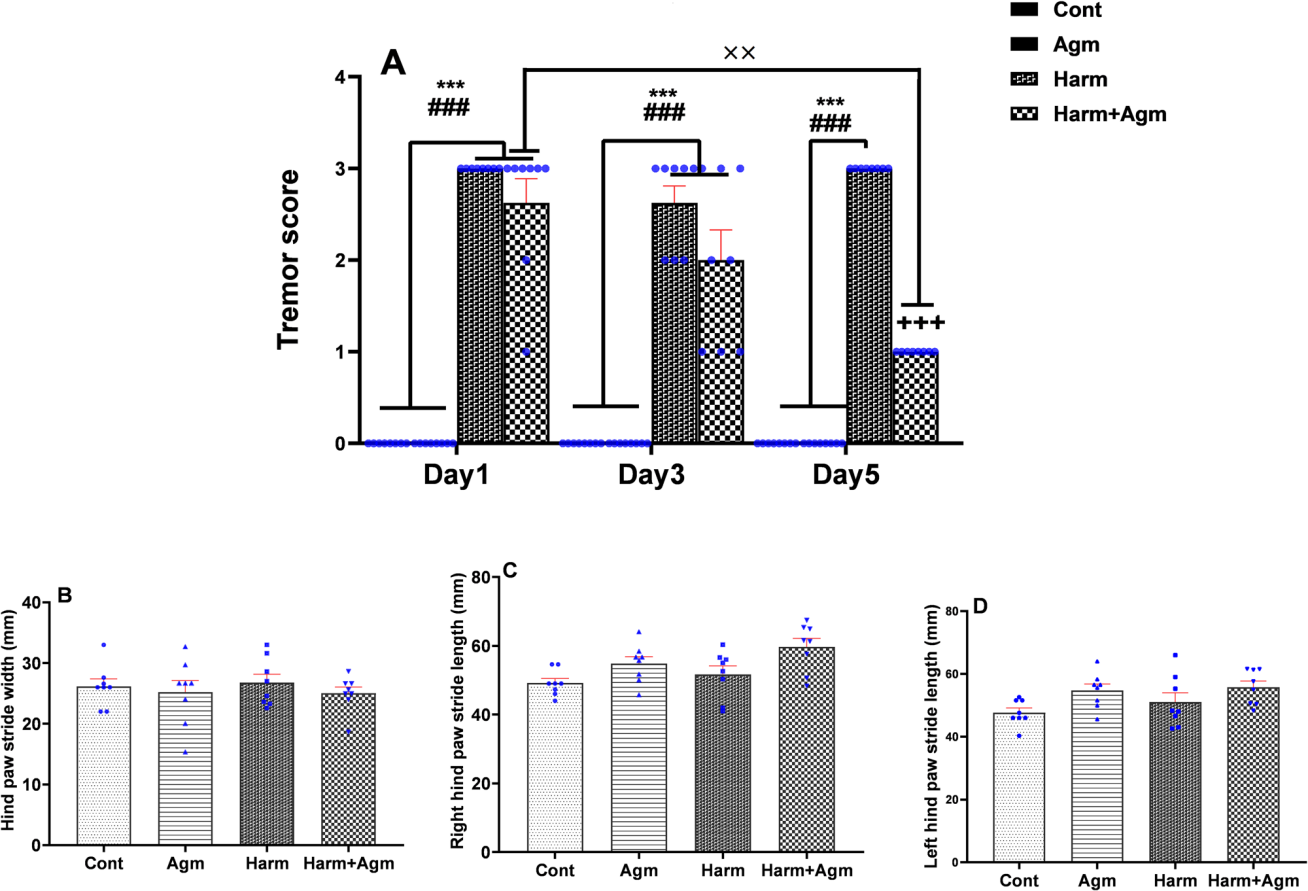
Tremor score rating showed that harmaline significantly increased tremor scores compared to scores noted in the control and agmatine groups in Days 1, 3, and 5. The harmaline + agmatine group showed significantly lower tremor scores in comparison with scores seen in the harmaline group on Day 5. In addition, the tremor scores were significantly less in Day 5 compared to Day 1 within the harmaline + agmatine group (A). There was no significant difference in hind paw stride width (B), right hind paw stride length (C), and left hind paw stride length (D) between the groups. All data are expressed as the mean ± SEM. ***p < 0.001 compared to the control group; ^###^ *p* < 0.001 compared to the agmatine group; ^+++^ *p *< 0.001 compared to the harmaline group. ^××^ *p *< 0.01 between days within the harmaline + agmatine group. Agm, agmatine; Cont, control; Harm, harmaline.

The repeated‐measures two‐way ANOVA of the tremor scores showed a significant interaction of group × day (*F*(6, 56) = 12.8, *p* = 0.001; Figure 2A). Analyses of the tremor scores revealed that harmaline injection resulted in a significant increase in tremor in Days 1, 3, and 5 of the study, as the harmaline and harmaline + agmatine groups showed significantly higher tremor scores than the control and agmatine groups (*p* < 0.001). On Day 5, the harmaline + agmatine group demonstrated a significantly lower tremor score compared to the harmaline group (*p* < 0.001), suggesting that agmatine treatment attenuated the harmaline‐induced tremor at Day 5 (Figure 2A). Moreover, the tremor scores on Day 5 were significantly less than on Day 1 within the harmaline + agmatine group (*p* < 0.01; Figure 2A). On the other hand, no significant differences were observed in hind paw stride width (one‐way ANOVA; *F*(3, 28) = 0.32, *p* = 0.8; Figure 2B) and length (Right: *F*(3, 28) = 4.5; Figure 2C; Left: *F*(3, 28) = 2.9; Figure 2D) between the study groups.

### 3.2 Effects of Agmatine on Locomotion and Anxiety‐Like Behavior Induced by Harmaline

**FIGURE 3:**
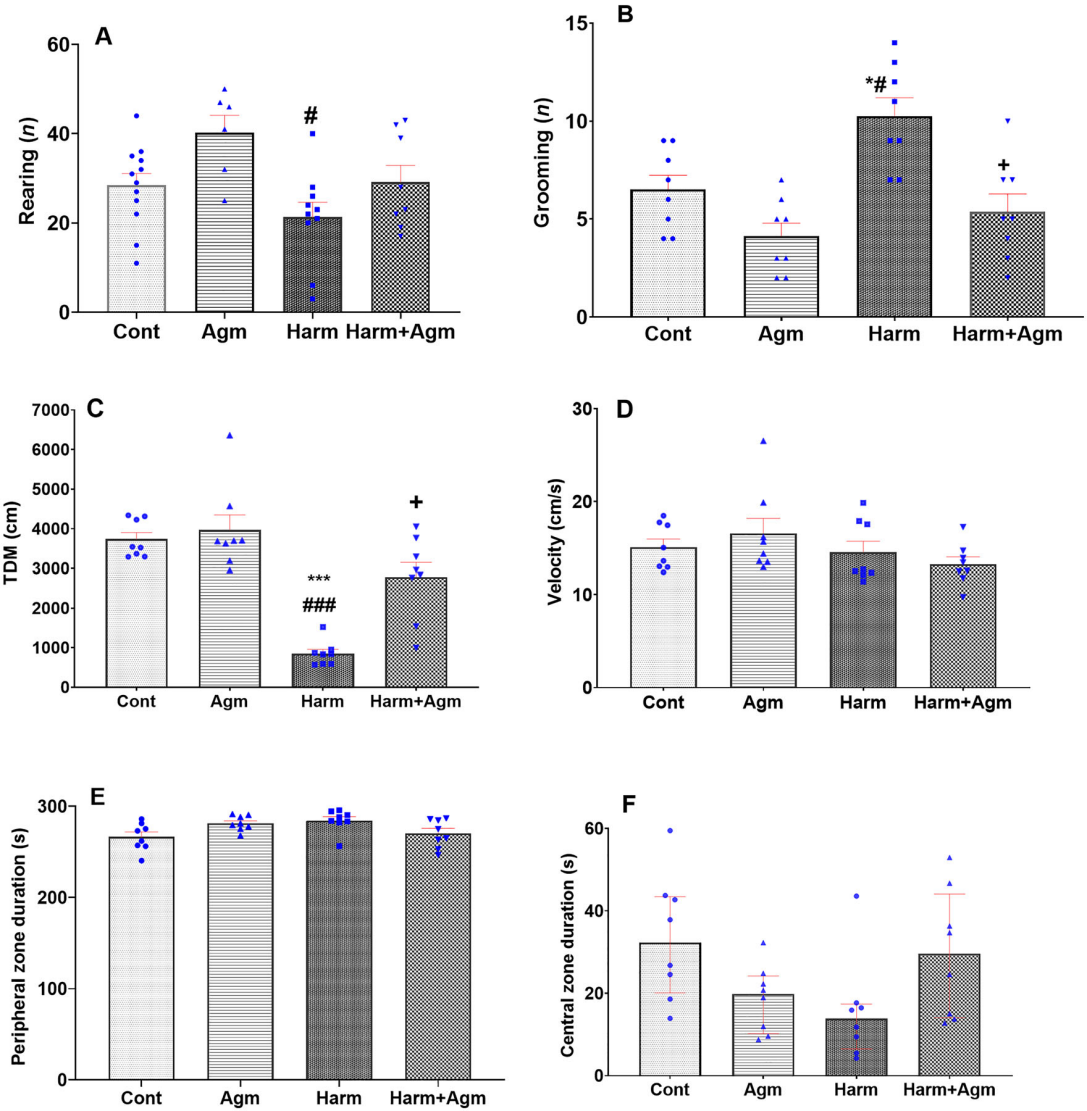
Open field test. Number of rearings (A), number of grooming events (B), total distance moved (C), velocity (D), time spent in the peripheral zone (E), and time spent in the central zone (F). The harmaline group had fewer rearings than the agmatine group, but more grooming events compared to the control and agmatine groups. Importantly, the harmaline + agmatine group showed reduced grooming compared to the harmaline group. The harmaline group also had lower TDM than control and agmatine groups, but the harmaline + agmatine group had higher TDM than the harmaline group, indicating that agmatine improved the harmaline‐induced locomotion impairment. No differences were found among groups in velocity, time spent in the peripheral zone time, or time spent in the central zone time. Data are expressed as the mean ± SEM in A, B, C, D, and E. Data are expressed as median with interquartile range in F. ***p < 0.001, *p < 0.05 compared to the control group; ^###^ *p* < 0.001, ^#^ *p* < 0.05 compared to the agmatine group; ^+^ *p* < 0.05 compared to the harmaline group. Agm, agmatine; Cont, control; Harm, harmaline; TDM, total distance moved.

The locomotion and anxiety‐like behaviors were assessed according to the open field test. Findings revealed that the harmaline group had a significantly lower number of rearings in comparison to that exhibited by the agmatine group (*F*(3, 32) = 4.4, *p* =  0.01; Figure 3A). Moreover, the harmaline group demonstrated a significantly higher number of grooming events compared to those seen in the control and agmatine groups (*F*(3, 28) = 10.4, *p* = 0.0001; Figure 3B). Importantly, the harmaline + agmatine group showed a significantly lower number of grooming events compared to the harmaline group (*p* < 0.05), suggesting that agmatine treatment reduced the anxiety‐like behavior in the harmaline group to a level comparable to the control and agmatine groups (Figure 3B). Furthermore, our findings indicated that the harmaline group had a significantly lower TDM compared to the control and agmatine groups (*F*(3, 28) = 25.2, *p* = 0.0001; Figure 3C); however, the harmaline + agmatine group showed a significantly higher TDM in comparison to the harmaline group (*p* < 0.05), highlighting that agmatine normalized the harmaline‐induced locomotion impairment to a level comparable to both the control and agmatine groups (Figure 3C). There were no differences among the groups in velocity (*F*(3, 28) = 1.3, *p* = 0.26; Figure 3D), time spent in the peripheral zone (*F*(3, 28) = 3.3, *p* = 0.03 with nonsignificant post hoc tests; Figure 3E), and time spent in the central zone (*F*(3, 28) = 3.39, *p* = 0.031 with nonsignificant post hoc tests; Figure 3F).

### 3.3 Harmaline and Agmatine Did Not Affect Balance and Muscle Strength

**FIGURE 4:**
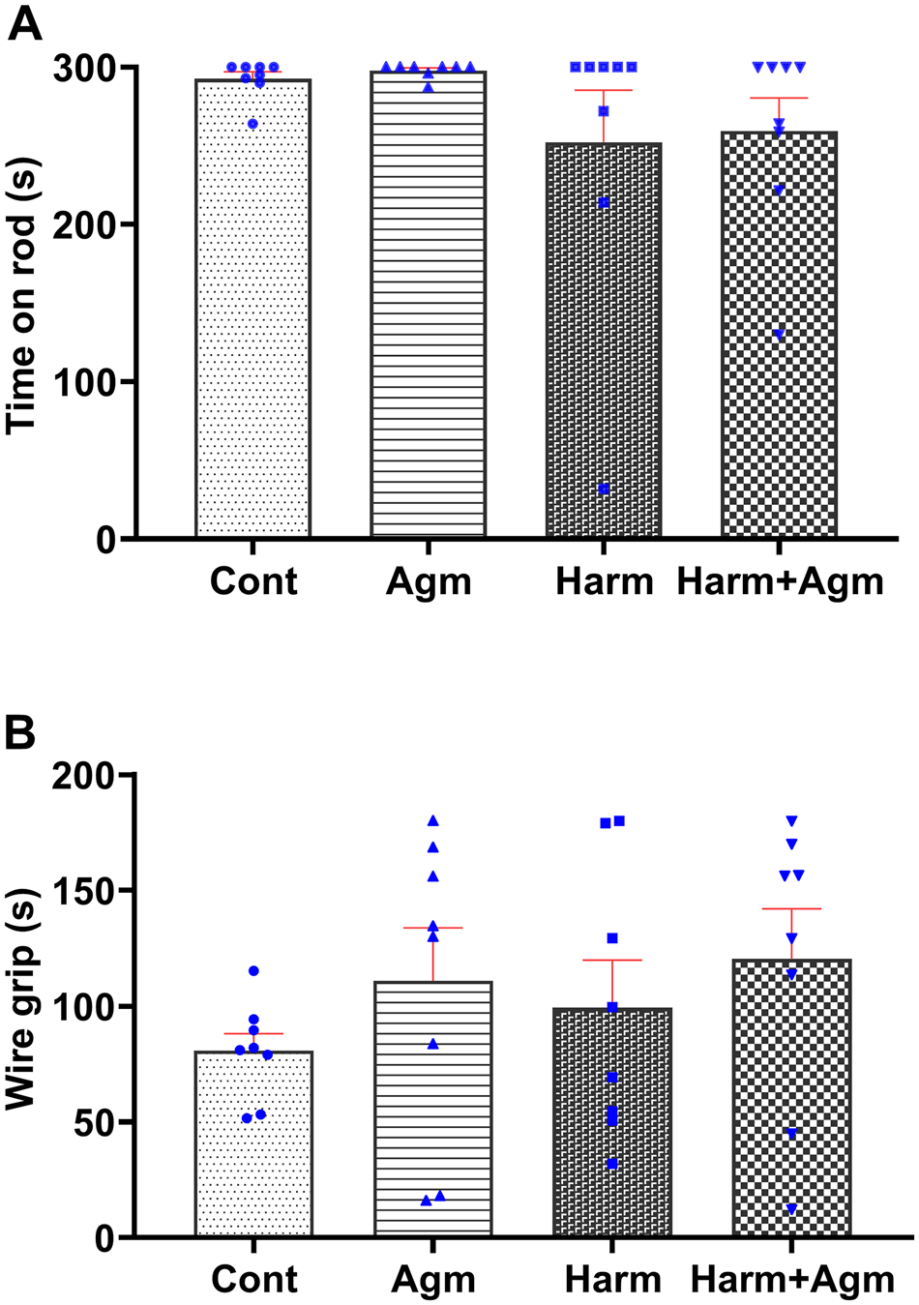
Rotarod performance test (A) and wire grip test (B) results indicate that harmaline did not significantly impact balance or muscle strength. Data are expressed as the mean ± SEM. Agm, agmatine; Cont, control; Harm, harmaline.

Evaluation of the balance (*F*(3, 28) = 1.3, *p* = 0.27; Figure 4A) and muscle strength (*F*(3, 28) = 0.7, *p* = 0.5; Figure 4B) according to the rotarod performance test and wire grip test showed that harmaline did not induce any significant alterations in these indices. No differences were observed between the four groups in either of the tests (Figure 4A,B).

### 3.4 Harmaline and Agmatine Did Not Affect Passive Avoidance Learning and Memory

**FIGURE 5:**
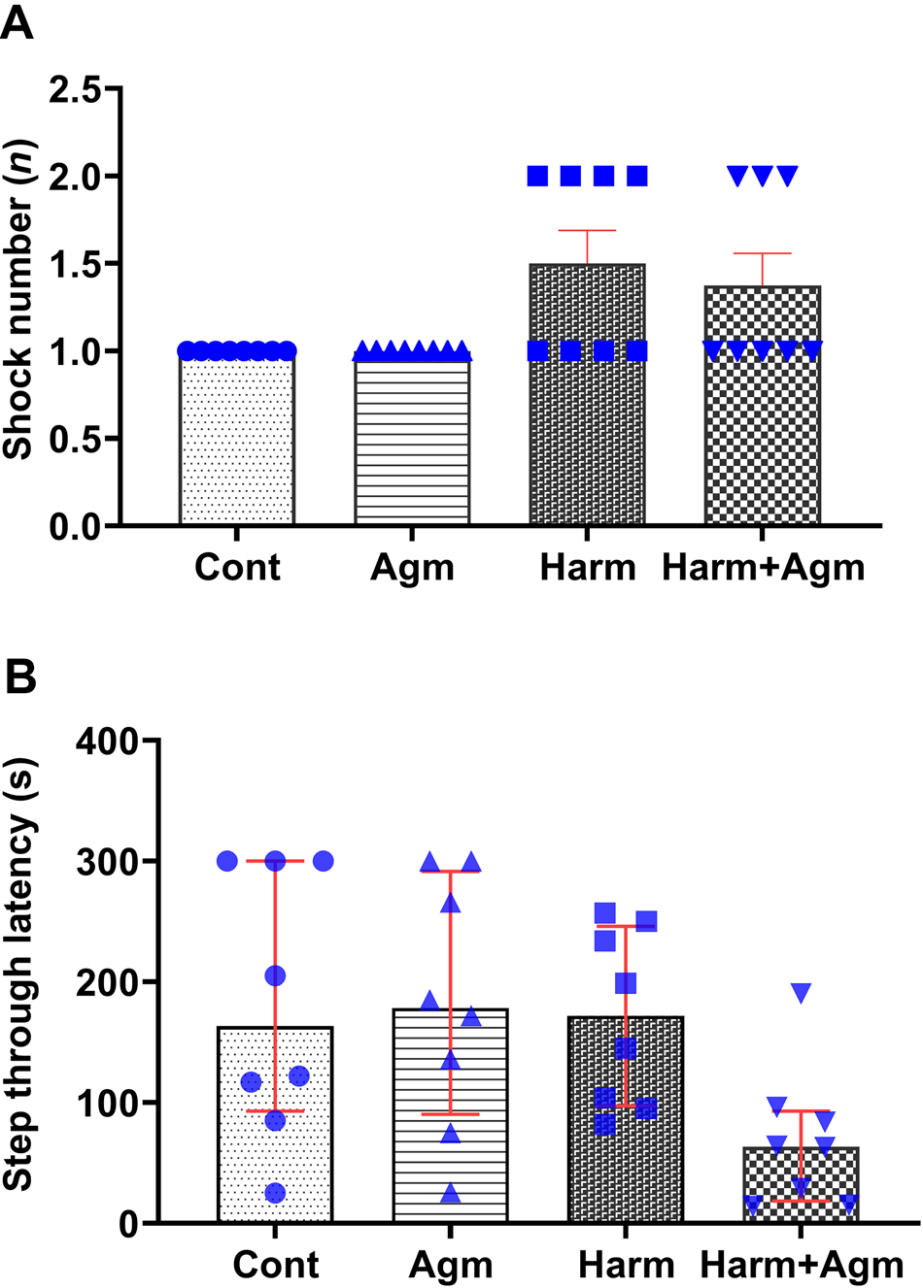
Passive avoidance learning (A) and memory (B). There were no significant differences in the shock number and step‐through latency between the groups. Data are expressed as the mean ± SEM in A, and as median with interquartile range in B. Agm, agmatine; Cont, control; Harm, harmaline.

Based on the findings of the passive avoidance test, there were no significant differences in the shock number (*F*(3, 28) = 3.8, *p* = 0.2; Figure 5A) and STL (*F*(3, 28) = 3.08, *p* = 0.043 with nonsignificant post hoc tests; Figure 5B) between the four experimental groups.

### 3.5 Agmatine Significantly Reduced the Harmaline‐Induced Overexpression of Lingo‐1

**FIGURE 6:**
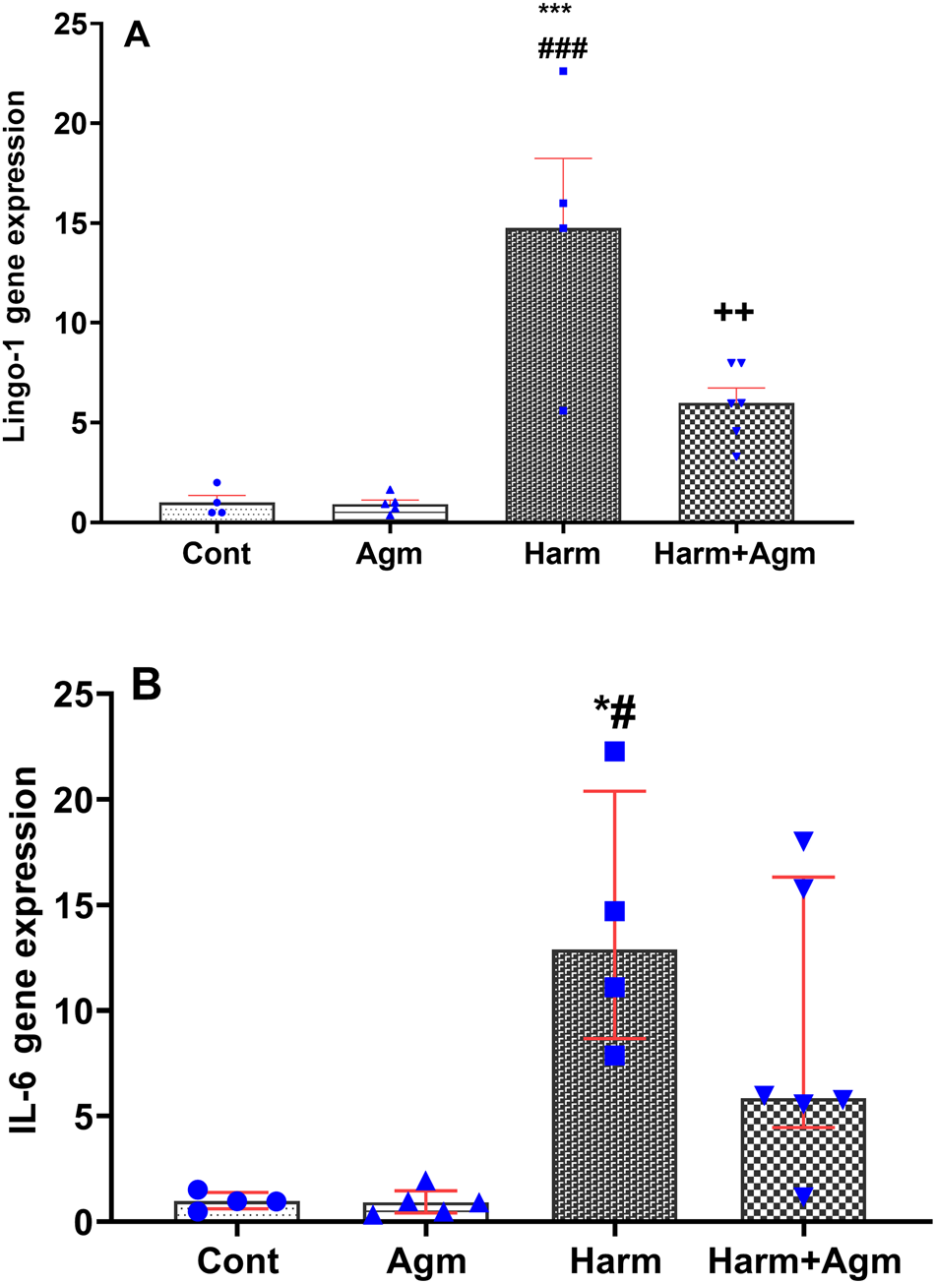
RT‐PCR test assessing the expression of Lingo‐1 (A) and IL‐6 (B). The harmaline group demonstrated higher levels of Lingo‐1 and IL‐6 expression than the control and agmatine groups. The Lingo‐1 expression was significantly lower in harmaline + agmatine group compared to the harmaline group. The IL‐6 expression in the harmaline + agmatine group was comparable to the control, but not significantly different compared to the harmaline group. Data are expressed as the mean ± SEM in A and as median with interquartile range in B. ***p < 0.001, *p < 0.05 compared to the control group; ^###^ *p* < 0.001, ^#^ *p *< 0.05 compared to the agmatine group; ^++^ *p* < 0.01 compared to the harmaline group. Agm, agmatine; Cont, control; Harm, harmaline.

Analyzing the expression of Lingo‐1 using real‐time PCR demonstrated that harmaline‐treated animals had a significantly higher expression of Lingo‐1 compared to the agmatine and control groups (*F*(3, 15) = 15.1, *p* = 0.0001; Figure 6A). Notably, the harmaline + agmatine group demonstrated a significantly lower level of Lingo‐1 expression compared to the harmaline group (*p* < 0.01), which was comparable to the control and agmatine groups (Figure 6A). These findings show that agmatine reduced the overexpression of Lingo‐1 in the harmaline‐induced animal model of ET.

### 3.6 Agmatine Attenuated the Harmaline‐Induced Overexpression of IL‐6

Using real‐time PCR, the harmaline group demonstrated an elevated level of IL‐6 compared to the control and agmatine groups (*F*(3, 15) = 7.8, *p* = 0.002; Figure 6B). On the other hand, IL‐6 expression in the harmaline + agmatine group did not differ significantly from that of the control and agmatine groups (Figure 6B). Although the difference between the harmaline and harmaline + agmatine groups was not significant, the values in the harmaline + agmatine group were comparable to the control and agmatine groups. These findings imply that agmatine could partially attenuate the overexpression of IL‐6 to a comparable level as the control groups.

## 4 Discussion

The key findings of this study demonstrate that agmatine treatment attenuates harmaline‐induced tremor and behavioral impairments in a rodent model of ET. Agmatine administration also attenuated the overexpression of Lingo‐1 and partially normalized IL‐6 overexpression induced by harmaline. Taken together, our results suggest that agmatine may confer neuroprotective effects in ET, potentially through modulating Lingo ‐1 and neuroinflammatory pathways.

Although agmatine has been studied as a neuroprotective agent in various neurodegenerative and neuropsychiatric disorders, its therapeutic potential in ET is less well explored. The tremor‐attenuating effect of agmatine observed herein is consistent with previous findings demonstrating its efficacy in the rat model of harmaline‐induced tremor in which agmatine reduced the tremor duration and intensity induced by harmaline, whereas no effects on locomotor activity were observed (Akman et al. 2021). Similarly, our findings revealed that while harmaline administration resulted in tremors on Days 1, 3, and 5, multiple doses of agmatine treatment resulted in the dampening of these tremors with significant tremor reduction by Day 5. Our data are indicative of the ability of agmatine to counter the neurotoxic effects of harmaline. Tremor induction by harmaline has been proposed to be due to the abnormal synchronized activation of glutamatergic climbing fibers in the olivocerebellar system (Handforth 2012). Therefore, the anti‐tremor effects of agmatine could arise from its known antagonism of glutamatergic NMDA receptors. On the other hand, activation of climbing fibers also elevates the levels of excitatory amino acids, nitric oxide (NO), and cyclic guanosine monophosphate (cGMP) in the cerebellum (Handforth 2012; G. Yang and Iadecola 1998), which are believed to take part in the progression of the tremors induced by harmaline. As agmatine is also recognized as an inhibitor of NOS (Piletz et al. 2013), reduction of NO production represents another potential mechanism contributing to its anti‐tremor actions.

In addition to the reduction of tremor, agmatine administration normalized certain harmaline‐induced behavioral abnormalities. Previous studies have shown that harmaline can reduce locomotor activity (Akman et al. 2021; Iseri et al. 2011; Kosmowska et al. 2016; Giacobbo et al. 2020). Consistent with these findings, we demonstrated that harmaline‐treated animals exhibited significantly lower TDM, which suggests a degree of impaired locomotion. Notably, agmatine ameliorated the locomotor deficits in harmaline‐treated animals, suggesting the role of agmatine in preserving functionality of neural circuitry controlling locomotion in the context of neurotoxic insult. Similar effects have been observed with other NMDA antagonist agents as evidenced by memantine's ability to alleviate harmaline‐induced deficits in locomotor activity (Iseri et al. 2011). Contradictory findings have been obtained regarding the effect of harmaline on inducing anxiety‐like behaviors, which have been attributed to dose effects (Hilber and Chapillon 2005; Mosaffa et al. 2021). In this study, we found that harmaline was associated with some anxiety‐like behaviors in the open field paradigm (i.e., increased grooming and decreased rearing), suggesting an anxiogenic effect, which is consistent with other studies using similar doses (Pirmoradi et al. 2024; Hilber and Chapillon 2005). Agmatine ameliorated the anxiety‐like behaviors caused by harmaline. The attenuation of anxiety is consistent with previous reports of agmatine conferring anxiolytic effects (Gong et al. 2006; Lavinsky, Arteni, and Netto 2003). Overall, our behavioral findings underscore the neuroprotective properties of agmatine.

Our study also provides novel evidence of the interaction between agmatine and the Lingo‐1 pathway as a key mechanism implicated in the amelioration of ET pathogenesis. Lingo‐1 overexpression has been reported in the cerebellar cortex of individuals with ET (Kuo et al. 2013). Our findings showed a heightened level of cerebellar Lingo‐1 expression in harmaline‐treated animals. Furthermore, our molecular findings demonstrated that agmatine administration suppressed the overexpression of Lingo‐1 induced by harmaline. This suggests that modulation of Lingo‐1 could serve as a mechanism underlying agmatine's protective effects against the neurotoxicity elicited by harmaline. This is particularly relevant given that Lingo‐1 antagonism has been proposed as a novel treatment approach for ET (Agundez et al. 2015). Lingo‐1 plays a negative regulatory role in both oligodendrocyte differentiation and myelination, which is mediated through distinct mechanisms that involve the activation of RhoA‐GTPase, as well as interactions with nerve growth factor and the tyrosine kinase A receptor (Mi et al. 2005; Lee et al. 2007). Inhibiting Lingo‐1 has been shown to affect remyelination in several animal models of central nervous system demyelination (Mi, Blake Pepinsky, and Cadavid 2013). Accordingly, anti‐Lingo‐1 antibodies are currently being investigated in various neurological diseases, including models of Alzheimer's disease (Y. Zhou et al. 2023; H. Yang et al. 2022) and multiple sclerosis (Tran et al. 2014). Our study is the initial exploration of the potential of agmatine in influencing Lingo‐1 expression, however, additional research is necessary to fully understand the exact interaction between agmatine and components of the Lingo‐1 pathway.

In addition, our findings revealed that agmatine treatment partially attenuated harmaline‐induced elevations in IL‐6 levels to a level comparable to the control groups. IL‐6 is a key proinflammatory cytokine involved in neurotoxic processes, and our findings imply that alongside Lingo‐1 modulation, regulation of neuroinflammation could comprise an additional mechanism through which agmatine protects against harmaline toxicity since agmatine has been shown to exhibit anti‐inflammatory and anti‐oxidative actions (J. M. Kim et al. 2016; El‐Sayed et al. 2019; Chai et al. 2016). Exploration of additional inflammatory mediators that are also known to be increased in ET (Muruzheva, Ivleva, et al. 2022) would provide further insight into agmatine's anti‐inflammatory effects in this context.

Besides the underlying mechanisms explored in this study, several studies have investigated potential mechanisms underlying the neuroprotective effects of agmatine. Agmatine has been shown to enhance neuroplasticity by regulating the levels of brain‐derived neurotrophic factor (BDNF) (Gawali et al. 2017; Bağcı et al. 2017). Agmatine is also known to inhibit NOS, thereby modulating nitrergic signalling (Gawali et al. 2016). Moreover, inhibition of glutamatergic NMDA receptors and activation of GABA_A_ receptors are involved in the effects of agmatine (Neis et al. 2020). These mechanisms should be explored further to determine whether they play a role in agmatine's neuroprotective properties in ET.

Some limitations must be acknowledged regarding this study. First, as ET is more common in males and appears at an earlier age when compared to the onset age in females (Song et al. 2021), we focused on male animals in this study, which restricts the generalization of findings across sexes. Thus, as neurodegenerative processes could differ according to sex and therefore call for more precise approaches in the management of ET, similar studies should be conducted in females (Bianco, Antonacci, and Liguori 2023). Second, in this study, the anxiogenic effects of harmaline were only explored through an open field test; however, utilizing more specific tests (e.g., elevated plus maze) could provide more accurate insights into the effects of harmaline (and agmatine) on anxiety‐like behavior. Third, while molecular analyses focused specifically on Lingo‐1 and IL‐6 within the cerebellum, assessment of these and additional markers in other relevant regions could offer greater mechanistic insight. Fourth, due to the exploratory nature of our study, we allocated our resources to thoroughly assess the effects of agmatine across multiple behavioral and molecular parameters. Therefore, a positive control to compare the effects of agmatine with other known interventions was not performed. We recognize the value of including a positive control in future studies that will focus on the superiority (or non‐inferiority) of agmatine compared to conventional treatments. Finally, while the harmaline‐induced tremor serves as a widely utilized and well‐established rodent model for ET, it is important to note that this model represents an acute and temporary condition that stands in contrast to the chronic and neurodegenerative nature of ET in humans, suggesting a greater complexity of disease mechanisms in ET. Regardless, the harmaline model of ET is a standard preclinical model and has provided insight into mechanisms underlying the disorder.

## 5 Conclusions

In summary, this is the first study demonstrating that agmatine treatment confers protective effects against harmaline‐induced neurotoxicity, potentially by normalizing elevated Lingo‐1 expression and neuroinflammatory processes. These preclinical findings suggest that agmatine merits further investigation as a prospective therapeutic agent for ET. Elucidating the precise mechanisms underlying agmatine's modulation of pathways implicated in ET would substantiate its promise as a novel intervention.

## Author Contributions

**Zeynab Pirmoradi**: investigation, methodology, data curation. **Zahra Esmaili**: investigation, methodology, data curation. **Mohsen Nakhaie**: investigation, methodology, data curation. **Kristi A. Kohlmeier**: writing–original draft, writing–review and editing, supervision. **Mohammad Shabani**: conceptualization, methodology, data curation, formal analysis, visualization, validation. **Moazamehosadat Razavinasab**: conceptualization, methodology, data curation, supervision, validation. **Mehran Ilaghi**: investigation, methodology, writing–original draft, writing–review and editing, visualization.

## Conflicts of Interest

The authors declare no conflicts of interest.

### Peer Review

The peer review history for this article is available at https://publons.com/publon/10.1002/brb3.70241.

## Data Availability

The data that support the findings of this study are available from the corresponding author upon reasonable request.
